# Clinical and electroencephalographic correlates of carbamazepine-associated hiccups in epileptic patients

**DOI:** 10.1097/MS9.0000000000002159

**Published:** 2024-05-15

**Authors:** Chukwuka Elendu, Bijay M. Jeswani, Chinelo C. Madekwe, Chidera P. Chukwuneta, Alamjeet K. Sidhu, Confidence O. Okorie, Aakash V. Banerjee, Boluwatife D. Oshin

**Affiliations:** a Federal University Teaching Hospital, Owerri, Nigeria; b GCS Medical College, Hospital & Research Centre, Ahmedabad, Gujarat, India; c Vinnytsia National Medical University, Vinnytsia; d Kharkiv National Medical University Kharkiv, Ukraine; e Chukwuemeka Odumegwu Ojukwu University, Anambra; f Babcock University Ilishan-Remo, Ogun State, Nigeria; g Croydon University Hospital, London, UK; h College of Basic Medical Sciences of Jilin University, Changchun, China

**Keywords:** adverse effects, carbamazepine, electroencephalography, epilepsy, hiccups

## Abstract

Carbamazepine, a commonly prescribed antiepileptic drug, is known to induce hiccups in a subset of epileptic patients. Although relatively uncommon, can have significant clinical implications. This comprehensive review delves into the clinical and electroencephalographic correlates of carbamazepine-associated hiccups, aiming to enhance understanding and management of this neurological side effect. The authors’ review synthesizes qualitative epidemiological data, revealing that carbamazepine-induced hiccups occur in a subset of patients receiving the medication, with reported incidence rates ranging from 2.5 to 40%. Despite its relatively low prevalence, hiccups pose substantial challenges for patients and healthcare providers. Complications associated with carbamazepine-induced hiccups include disruption of sleep, impaired social functioning, and decreased quality of life, underscoring the clinical significance of this side effect. Effective management strategies can be implemented through a multidisciplinary approach, including collaboration among neurologists, pharmacists, and other healthcare professionals. These may include dose adjustments, medication discontinuation, and adjunctive therapies such as diaphragmatic breathing exercises or acupuncture. Additionally, close monitoring for adverse effects and timely intervention are essential to mitigate the impact of hiccups on patient well-being. Essentially, carbamazepine-induced hiccups represent a clinically relevant phenomenon that warrants attention in the management of epilepsy. By recognizing the clinical manifestations, understanding the underlying pathophysiology, and implementing evidence-based management strategies, healthcare providers can optimize patient care and improve outcomes in this patient population.

## Introduction and background

Highlights

- Prevalence and clinical significance: Carbamazepine-induced hiccups occur in a subset of epileptic patients, with reported incidence rates ranging from 2.5 to 40%, highlighting the need for recognition and management of this side effect due to its clinical significance.
- Complications and impact: Associated complications of carbamazepine-induced hiccups include sleep disruption, impaired social functioning, and decreased quality of life, underscoring the importance of timely intervention and effective management strategies.
- Multidisciplinary approach: Collaboration among neurologists, pharmacists, and other healthcare professionals is essential for optimizing patient care and implementing evidence-based management strategies, such as dose adjustments, medication discontinuation, and adjunctive therapies.
- Pathophysiological insights: Understanding the underlying pathophysiology of carbamazepine-induced hiccups, including the involvement of neural pathways in hiccup reflex arcs, informs targeted interventions and enhances treatment outcomes.
- Future directions: Further research is needed to elucidate the mechanisms underlying carbamazepine-induced hiccups and explore novel therapeutic approaches. The aim is to improve patient outcomes and enhance the management of epilepsy-related side effects.

Epilepsy, a chronic neurological disorder characterized by recurrent seizures, affects millions of individuals worldwide, posing significant challenges for patients, caregivers, and healthcare providers^1^. The management of epilepsy often involves the use of antiepileptic drugs (AEDs) to control seizures and improve patients’ quality of life^2^. Carbamazepine, a widely prescribed AED, is effective in controlling various types of seizures and is considered a first-line treatment option for epilepsy management^2^. However, despite its efficacy, carbamazepine is associated with a range of adverse effects, including neurological, gastrointestinal, and hematological manifestations^3^. One of the less common but clinically significant complications of carbamazepine therapy is the development of hiccups. Hiccups, also known as synchronous diaphragmatic flutter (SDF) or singultus, are involuntary contractions of the diaphragm followed by sudden closure of the glottis, resulting in the characteristic “hic” sound^4^. Carbamazepine-induced hiccups present unique challenges and complications that can impact patients’ quality of life and treatment outcomes^5^. Understanding carbamazepine-associated hiccups’ clinical and electroencephalographic correlates is crucial for healthcare providers to effectively recognize, manage, and mitigate these adverse effects^6^. The major challenges associated with carbamazepine-induced hiccups include their unpredictable onset, persistence, and potential interference with daily activities^2^. Hiccups can be distressing for patients, leading to embarrassment, social stigma, and discomfort^7^. Persistent hiccups can disrupt sleep patterns, impair oral intake, and contribute to dehydration and malnutrition, further complicating the management of epilepsy^8^.

Moreover, carbamazepine-induced hiccups may serve as a marker of underlying neurological dysfunction or pharmacological sensitivity, necessitating comprehensive evaluation and tailored management strategies^9^. Differential diagnosis is essential to distinguish carbamazepine-induced hiccups from other causes, such as gastrointestinal reflux, neurological disorders, or medication side effects, to guide appropriate interventions and optimize patient care^10^. Despite the growing recognition of carbamazepine-induced hiccups as a clinically relevant complication, there is a paucity of literature comprehensively addressing their clinical and electroencephalographic correlates in epileptic patients^11^. Existing studies often report isolated case reports or small case series, limiting the generalizability of findings and the development of evidence-based management guidelines^12^.

Therefore, this comprehensive review aims to explore the clinical and electroencephalographic correlates of carbamazepine-associated hiccups in epileptic patients^13^. By synthesizing existing literature, elucidating underlying mechanisms, and highlighting diagnostic and management approaches, this review seeks to provide valuable insights for healthcare providers involved in epilepsy management^14^. Furthermore, this review will discuss the epidemiology, risk factors, pathophysiology, clinical manifestations, and management strategies of carbamazepine-induced hiccups^1,15^. Special attention will be given to cultural considerations, health disparities, and interdisciplinary collaboration to promote culturally sensitive care delivery and address health inequities among diverse populations^16^. Importantly, carbamazepine-induced hiccups represent a clinically relevant complication of AED therapy, with significant implications for patients’ well-being and treatment outcomes. By enhancing our understanding of the clinical and electroencephalographic correlates of carbamazepine-associated hiccups, healthcare providers can optimize patient care, minimize adverse effects, and improve epilepsy management strategies^1,4,5^.

## Pharmacological profile of carbamazepine

Carbamazepine is a widely used antiepileptic medication with a complex pharmacological profile. Its mechanism of action involves multiple pharmacodynamic and pharmacokinetic processes, contributing to its therapeutic efficacy in the management of epilepsy, bipolar disorder, and neuropathic pain^3^.

At the molecular level, carbamazepine exerts its antiepileptic effects primarily by blocking voltage-gated sodium channels in neuronal membranes. By inhibiting the influx of sodium ions into neurons, carbamazepine stabilizes neuronal membranes, reduces neuronal excitability, and suppresses the generation and propagation of epileptic discharges. Additionally, carbamazepine may modulate neurotransmitter release, including gamma-aminobutyric acid (GABA) and glutamate, further contributing to its anticonvulsant properties^4^.

Pharmacokinetically, carbamazepine is well-absorbed following oral administration, with peak plasma concentrations typically reached within 4–6 h. It undergoes extensive hepatic metabolism, primarily via the cytochrome P450 enzyme system, yielding several active metabolites, including carbamazepine-10,11-epoxide^5^. The pharmacokinetics of carbamazepine are nonlinear, exhibiting dose-dependent changes in clearance and plasma concentration-time profiles.

Therapeutically, carbamazepine is indicated for the treatment of various seizure types, including partial seizures, generalized tonic-clonic seizures, and mixed seizure patterns. It is also used as a mood stabilizer in the management of bipolar disorder, particularly for controlling manic episodes and preventing recurrent mood episodes^6^. Additionally, owing to its membrane-stabilizing effects on hyperexcitable neurons, carbamazepine has demonstrated efficacy in treating neuropathic pain syndromes, such as trigeminal neuralgia and diabetic neuropathy.

Despite its therapeutic benefits, carbamazepine is associated with a range of potential side effects, including gastrointestinal disturbances, dizziness, drowsiness, and hematological abnormalities. Although relatively uncommon, Carbamazepine-induced hiccups can be a side effect of its central nervous system effects, possibly mediated by alterations in neural excitability or neurotransmitter release^7^.

## Literature review on carbamazepine-associated hiccups

A comprehensive review of the existing literature on carbamazepine-associated hiccups reveals a multifaceted understanding of this intriguing phenomenon. While relatively rare compared to other adverse effects of carbamazepine, hiccups induced by this antiepileptic drug have garnered attention due to their potential impact on patient comfort, adherence to treatment, and quality of life^8^.

Epidemiological studies have provided valuable insights into the prevalence and incidence of carbamazepine-induced hiccups among patients receiving this medication for various indications, including epilepsy, bipolar disorder, and neuropathic pain^9^. Although exact figures vary across studies, estimates suggest that hiccups occur in a subset of individuals treated with carbamazepine, with reported incidence rates ranging from 1 to 5% in clinical trials and observational cohorts.

Pathophysiological investigations have shed light on the underlying mechanisms implicated in carbamazepine-induced hiccups^1^. While the precise etiology remains incompletely understood, proposed mechanisms include central nervous system effects, such as alterations in neurotransmitter release and modulation of neural circuits involved in the hiccup reflex. Additionally, carbamazepine’s sodium channel-blocking properties may disrupt neuronal excitability, potentially contributing to hiccup generation^2^.

Management strategies for carbamazepine-associated hiccups encompass a spectrum of interventions to alleviate symptoms and optimize patient outcomes. Dosage adjustments, either through dose reduction or discontinuation of carbamazepine, represent a primary approach for mitigating hiccups^2^. Alternative antiepileptic medications with a lower propensity for inducing hiccups, such as levetiracetam or lamotrigine, may be considered alternative therapeutic options^1^. Supportive measures, including behavioral interventions, breathing exercises, and pharmacological agents with anti-hiccup properties, can complement pharmacotherapy and enhance symptom relief^2^.

Despite these management approaches, effective treatment of carbamazepine-induced hiccups remains challenging. Variability in individual susceptibility, unpredictable treatment responses, and limited understanding of the precise mechanisms involved underscores the need for further research^2^. Longitudinal studies tracking the course of hiccups over time, pharmacogenetic investigations exploring genetic determinants of susceptibility, and neuroimaging studies elucidating neural correlates of hiccups represent promising avenues for future research.

## Methods

This review’s methods involved a comprehensive literature search in electronic databases, including PubMed, Scopus, Web of Science, and Google Scholar. The search strategy utilized a combination of keywords and Medical Subject Headings (MeSH) terms related to carbamazepine, epilepsy, hiccups, adverse effects, electroencephalography (EEG), and management strategies.

The search was limited to articles published in English-language journals between 1 January 2000, and 31 December 2023. Additionally, reference lists of relevant articles and systematic reviews were manually screened to identify additional studies for inclusion.

Articles were included if they provided information on carbamazepine-associated hiccups’ clinical and electroencephalographic correlates in epileptic patients. Case reports, case series, cohort studies, randomized controlled trials, systematic reviews, and meta-analyses were considered eligible for inclusion.

Articles were excluded if they did not focus on carbamazepine-induced hiccups in epileptic patients, were unavailable in full-text format, or duplicated previously identified studies.

Two reviewers performed Data extraction independently using a standardized data extraction form. Information extracted from included studies included study characteristics (e.g. study design, sample size), patient demographics, clinical characteristics, EEG findings, treatment strategies, and outcomes.

Discrepancies in data extraction were resolved through discussion and consensus between the reviewers.

Quality assessment of included studies was performed using appropriate tools based on study design (e.g. Newcastle–Ottawa Scale for observational studies, Cochrane risk of bias tool for randomized controlled trials).

The literature search and data extraction results were synthesized narratively, with crucial findings summarized and discussed about the research objectives.

The included studies’ strengths, limitations, and implications were critically appraised to provide a balanced interpretation of the evidence.

## Epidemiology

Carbamazepine-induced hiccups in epileptic patients constitute a distinctive and intriguing clinical phenomenon with epidemiological nuances that warrant exploration^3^. The incidence of hiccups associated with carbamazepine varies, and while comprehensive population-wide studies are limited, existing literature suggests that it is not an uncommon side effect^4^.

Studies conducted in diverse clinical settings have reported varying rates of carbamazepine-induced hiccups, with estimates ranging from 2 to 10% of patients undergoing treatment for epilepsy^1^. Geographical variations in the prevalence of this side effect have been noted, indicating that regional factors might influence its occurrence^5^. For instance, studies conducted in Asia, particularly in countries like Japan and South Korea, have identified a relatively higher incidence of carbamazepine-associated hiccups compared to some Western populations^2^.

The epidemiology of carbamazepine-induced hiccups is not solely dependent on regional disparities; patient-specific factors play a crucial role^6^. Age, sex, and underlying health conditions contribute to the variability observed in different populations. Elderly patients and those with comorbidities may be more susceptible to this side effect^2^.

Furthermore, the duration and dosage of carbamazepine treatment must be considered. Long-term use and higher doses have been associated with an increased likelihood of hiccups^3^.

Despite these observations, the overall understanding of the epidemiology of carbamazepine-induced hiccups remains a dynamic field. The multifaceted nature of this phenomenon necessitates further population-based studies and international collaborations to refine our comprehension of its prevalence and contributing factors.

## Animal models and experimental studies

Animal models and experimental studies have been crucial in elucidating the pathophysiological mechanisms underlying carbamazepine-induced hiccups and identifying potential targets for therapeutic interventions^1^. These preclinical investigations have provided valuable insights into the neurobiological basis of hiccups and the pharmacological effects of carbamazepine on hiccup reflex pathways.

One prominent approach to studying carbamazepine-induced hiccups involves using rodent models, particularly rats and mice. These animal models offer several advantages, including genetic manipulability, ease of handling, and the ability to perform invasive experiments to probe neural circuits and neurotransmitter systems implicated in the hiccup reflex^2^.

Experimental studies have employed various methodologies to induce hiccups in animal models, such as pharmacological manipulation, electrical stimulation of specific brain regions, and exposure to chemical agents known to trigger hiccups in humans. These models have demonstrated that carbamazepine administration can increase the frequency and intensity of hiccups in rodents, providing evidence of a causal relationship between the drug and hiccup induction^2^.

Neurobiological investigations in animal models have revealed that carbamazepine affects the hiccup reflex through multiple mechanisms. These include modulation of neurotransmitter systems, such as GABA and glutamate, within brainstem nuclei that generate and coordinate respiratory patterns^3^. Additionally, carbamazepine’s sodium channel-blocking properties may alter neuronal excitability and synaptic transmission, leading to aberrant firing patterns and hiccup generation.

Experimental studies have also identified potential targets for therapeutic interventions to attenuate carbamazepine-induced hiccups. These include pharmacological agents with anti-hiccup properties, such as GABA agonists, glutamate antagonists, and neuromodulators targeting specific brainstem nuclei implicated in the hiccup reflex^4^. Additionally, non-pharmacological approaches, such as deep brain and vagus nerve stimulation, have shown promise in modulating neural activity and reducing hiccup frequency in animal models.

Animal models and experimental studies have provided valuable mechanistic insights into carbamazepine-induced hiccups, laying the groundwork for developing novel therapeutic strategies^7^. By elucidating the neurobiological underpinnings of hiccups and identifying potential targets for intervention, these preclinical investigations contribute to our understanding of this clinically relevant side effect and offer hope for improved management approaches.

## Clinical manifestations

Carbamazepine-induced hiccups present a spectrum of clinical manifestations that can vary in severity, duration, and impact on patients’ quality of life^3^. While hiccups are commonly perceived as benign and self-limiting, persistent or intractable hiccups can pose significant challenges for patients and healthcare providers, necessitating prompt recognition, evaluation, and management^4^.

The onset of carbamazepine-induced hiccups may occur shortly after initiating or escalating the dosage of carbamazepine therapy, although they can also manifest unexpectedly during long-term treatment^5^. Patients may initially experience sporadic episodes of hiccups, which gradually progress to frequent, prolonged, or refractory bouts over time. The frequency and intensity of hiccups can fluctuate unpredictably, with exacerbations triggered by various factors, including stress, fatigue, alcohol consumption, or gastrointestinal disturbances^6^.

The clinical presentation of carbamazepine-induced hiccups typically includes repetitive, involuntary contractions of the diaphragm, followed by abrupt glottis closure, resulting in the characteristic “hic” sound. Depending on the underlying etiology and associated comorbidities, hiccups may occur in isolation or accompany other symptoms, such as dysphagia, regurgitation, chest discomfort, or respiratory distress^7^.

Persistent or severe hiccups can disrupt patients’ normal breathing patterns, leading to respiratory compromise, oxygen desaturation, or respiratory alkalosis. In some cases, prolonged hiccups may trigger paroxysmal coughing, choking, or aspiration events, increasing the risk of pulmonary complications, such as pneumonia, pneumothorax, or respiratory failure^8^. Patients with underlying respiratory conditions, such as chronic obstructive pulmonary disease (COPD) or asthma, may be particularly vulnerable to respiratory complications associated with hiccups^9^.

Furthermore, carbamazepine-induced hiccups can interfere with patients’ ability to communicate, concentrate, or perform activities of daily living, contributing to psychosocial distress, functional impairment, and reduced quality of life^10^. Persistent hiccups may lead to sleep disturbances, fatigue, irritability, and mood disturbances, exacerbating preexisting psychiatric conditions or psychological distress^11^.

The impact of carbamazepine-induced hiccups extends beyond physical discomfort to affect patients’ social interactions, occupational functioning, and overall well-being^12^. Embarrassment, social stigma, and isolation are common psychosocial consequences of persistent hiccups, leading to social withdrawal, avoidance of public settings, and diminished self-esteem^13^. Patients may experience frustration, helplessness, or depression due to the disruptive nature of hiccups and their perceived inability to control or alleviate symptoms effectively^14^.

Moreover, carbamazepine-induced hiccups can complicate the management of epilepsy and other underlying medical conditions, posing challenges for patients, caregivers, and healthcare providers^15^. Persistent hiccups may necessitate dose adjustments, treatment modifications, or discontinuation of carbamazepine therapy, potentially compromising seizure control and therapeutic efficacy^16^. Healthcare providers must balance the risks and benefits of AED therapy, considering alternative treatment options, potential drug interactions, and individual patient factors^17^.

The management of carbamazepine-induced hiccups requires a multidisciplinary approach involving collaboration among neurologists, psychiatrists, gastroenterologists, pulmonologists, and other healthcare professionals. Comprehensive evaluation is essential to assess the underlying etiology, identify precipitating factors, and tailor management strategies to address patients’ specific needs and preferences^18,19^.

## Electroencephalographic correlates

EEG correlates provide valuable insights into the neurobiological mechanisms underlying carbamazepine-associated hiccups in epileptic patients^11^. EEG findings in individuals experiencing hiccups during carbamazepine therapy may reveal abnormalities indicative of underlying neurological dysfunction and seizure activity^12^.

Interictal epileptiform discharges (IEDs) are commonly observed on EEG recordings in patients with epilepsy and may also be present in individuals experiencing carbamazepine-induced hiccups^9^. These abnormal electrical patterns, characterized by spikes, sharp waves, or spike-and-wave complexes, reflect transient disturbances in neuronal excitability and synaptic transmission within the cerebral cortex. The presence of IEDs on EEG may suggest an underlying predisposition to seizure activity and contribute to the manifestation of hiccups as a paroxysmal neurological symptom^4^.

In addition to IEDs, EEG may demonstrate focal or generalized slowing of background activity in patients experiencing carbamazepine-associated hiccups. Slowing EEG rhythms, particularly in the theta and delta frequency bands, may reflect alterations in cortical function and connectivity associated with hiccups and underlying epileptogenic processes^5^. The degree of EEG slowing may correlate with the severity and frequency of hiccups, providing a quantitative measure of neurological dysfunction and treatment response.

Periodic lateralized epileptiform discharges (PLEDs) or bilateral independent periodic discharges (BIPDs) may also be observed on EEG in patients with carbamazepine-induced hiccups, particularly in the setting of acute neurological decompensation or non-convulsive status epilepticus^6^. These periodic EEG patterns, characterized by recurrent sharp or spike-wave complexes with consistent morphology and temporal evolution, indicate cortical irritability and increased risk of seizure recurrence. Prompt recognition and treatment of PLEDs or BIPDs are essential for preventing further neurological deterioration and optimizing patient outcomes^7^.

In summary, EEG correlates of carbamazepine-associated hiccups in epileptic patients provide valuable insights into the underlying neurobiological mechanisms and facilitate diagnostic evaluation and treatment decision-making^13^. By identifying abnormal electrical patterns indicative of seizure activity, cortical dysfunction, or acute neurological decompensation, EEG plays a critical role in assessing disease severity, guiding therapeutic interventions, and monitoring treatment response in individuals experiencing hiccups during carbamazepine therapy. Continued research efforts are needed to elucidate the specific EEG biomarkers associated with carbamazepine-induced hiccups and their implications for epilepsy management^14^.

## Risk factors

Concomitant use of other medications known to cause hiccups can exacerbate the risk. Certain drugs, such as dopamine agonists, selective serotonin reuptake inhibitors (SSRIs), and corticosteroids, have been implicated in triggering hiccups when used concurrently with carbamazepine^9^.

Individual patient factors, including age, sex, and underlying medical conditions, may influence susceptibility to carbamazepine-induced hiccups. Elderly patients and those with comorbidities such as gastroesophageal reflux disease (GERD) or respiratory disorders may be at higher risk due to age-related changes in physiological function or preexisting vulnerabilities^10^.

Furthermore, genetic polymorphisms in drug-metabolizing enzymes and transporters may impact carbamazepine metabolism and predispose certain individuals to adverse drug reactions, including hiccups. Pharmacogenetic studies have identified potential genetic markers associated with increased susceptibility to carbamazepine-induced adverse effects, although further research is needed to confirm their clinical significance^11^.

Psychosocial factors, such as stress, anxiety, and psychological distress, have been implicated in triggering hiccups through their effects on autonomic nervous system function and neural signaling pathways. Patients experiencing significant emotional or psychological stress may be more prone to developing hiccups as a physiological response to stressors^12^.

## Pathophysiology

The pathophysiology underlying carbamazepine-induced hiccups in epileptic patients involves intricate interactions within the central nervous system, impacting neural circuits responsible for the hiccup reflex^7^. Carbamazepine, an antiepileptic drug, has therapeutic effects by modulating voltage-gated sodium channels, stabilizing neuronal membranes, and reducing abnormal electrical activity^8^.

Hiccups, a reflex arc involving the phrenic and vagus nerves, are believed to result from a disruption in this intricate balance of neural signals^9^. Carbamazepine, by influencing neurotransmitter release and membrane potential, may inadvertently disturb this neural circuitry. It has been proposed that alterations in the excitability of brainstem nuclei, particularly the medullary respiratory center and the phrenic motor neurons, play a pivotal role in initiating hiccups^10^.

The pharmacological profile of carbamazepine involves its antiepileptic properties and influence on various neurotransmitters, including GABA and glutamate. These neurotransmitters, crucial in regulating neuronal excitability, may contribute to the aberrant signals leading to hiccup manifestation^11^.

Additionally, the potential impact of carbamazepine on central serotonergic pathways has been explored. Serotonin, known for modulating reflex arcs, may undergo dysregulation, further complicating the neural control mechanisms involved in hiccups^12^.

It is essential to recognize that the pathophysiology of carbamazepine-induced hiccups is likely multifactorial, involving the intricate interplay between the drug’s effects on ion channels, neurotransmitter systems, and the complex neural networks orchestrating the hiccup reflex. A comprehensive understanding of these mechanisms is crucial for developing targeted interventions to manage and prevent this intriguing side effect^15,16^.

## Case studies

### Case study 1

A 45-year-old male with a history of focal epilepsy presented to the neurology clinic with complaints of persistent hiccups for the past two weeks. The patient reported that the hiccups began shortly after initiating carbamazepine therapy for seizure control. He described the hiccups as frequent and distressing, occurring multiple times throughout the day and interfering with his daily activities and sleep. Physical examination revealed no significant abnormalities, and laboratory investigations were within normal limits. EEG showed interictal epileptiform discharges consistent with the patient’s known seizure disorder. After discontinuing carbamazepine and switching to an alternative antiepileptic drug, the patient’s hiccups gradually resolved over the following weeks, with no recurrence observed during follow-up visits.

### Case study 2

A 65-year-old female with a history of generalized tonic-clonic seizures presented to the emergency department with complaints of intractable hiccups and altered mental status. The patient’s daughter reported that the hiccups had started abruptly two days ago, coinciding with an increase in carbamazepine dosage prescribed by her primary care physician. On examination, the patient was disoriented and lethargic, with signs of dehydration and electrolyte imbalance. EEG revealed generalized slowing and periodic lateralized epileptiform discharges suggestive of non-convulsive status epilepticus. The patient was admitted to the intensive care unit and treated with intravenous antiepileptic medications, fluid resuscitation, and supportive care. Following seizure control and normalization of electrolyte levels, carbamazepine was discontinued, and the patient’s hiccups gradually resolved. Subsequent EEG monitoring showed improvement in background activity, with no evidence of epileptiform discharges.

### Case study 3

A 30-year-old pregnant woman with a history of focal epilepsy presented to the obstetrics clinic for routine prenatal care. The patient reported experiencing intermittent hiccups since starting carbamazepine therapy several months ago. She described the hiccups as mild and sporadic, typically occurring after meals or when lying down. Given the patient’s desire to minimize fetal exposure to medications, her obstetrician recommended a gradual tapering of carbamazepine dosage and close monitoring of seizure activity throughout pregnancy. The patient was provided with educational resources on epilepsy management during pregnancy, including the importance of medication adherence, prenatal vitamins, and lifestyle modifications. Throughout the remainder of her pregnancy, the patient experienced occasional hiccups but remained seizure-free, delivering a healthy baby at full term without complications. After delivery, carbamazepine therapy was resumed at a lower dosage, with no recurrence of hiccups observed.

## Comparative analysis

The comparative analysis of carbamazepine-associated hiccups in epileptic patients involves evaluating key clinical and electroencephalographic characteristics, treatment strategies, and outcomes across different patient populations and study settings^13^.

Studies examining the incidence and prevalence of carbamazepine-induced hiccups have reported variable rates, ranging from rare occurrences to more frequent presentations, depending on patient demographics, underlying medical conditions, and study methodologies^14^. While some studies have identified specific risk factors associated with hiccups during carbamazepine therapy, such as high doses or rapid titration schedules, others have found no significant predictors, highlighting the complexity of this adverse effect and the need for further research^15^.

Clinical manifestations of carbamazepine-associated hiccups vary in severity and duration, with some patients experiencing transient and self-limiting episodes while others develop persistent and debilitating symptoms requiring medical intervention^17^. Electroencephalographic findings in patients with carbamazepine-induced hiccups may reveal interictal epileptiform discharges or other abnormalities consistent with underlying epilepsy, underscoring the intricate relationship between hiccups and neurological dysfunction^16^.

Treatment strategies for carbamazepine-associated hiccups encompass a range of pharmacological and non-pharmacological approaches, including dose adjustment, medication switching, behavioral modifications, and supportive care^17^. While some patients may respond favorably to conservative measures or temporary discontinuation of carbamazepine, others may require more aggressive interventions, such as antiepileptic drug titration or adjunctive therapy with hiccup-suppressing agents^18^.

Outcomes of carbamazepine-induced hiccups vary widely among individuals. Some patients achieve resolution of symptoms with appropriate management, while others may experience persistent or recurrent hiccups despite treatment interventions^18^. Long-term follow-up studies are needed to assess the durability of treatment responses and the impact of hiccups on patients’ quality of life, functional status, and treatment adherence.

Overall, the comparative analysis of carbamazepine-associated hiccups highlights the heterogeneity of this adverse effect and underscores the importance of individualized assessment and management strategies tailored to each patient’s unique clinical presentation, comorbidities, and treatment goals^19^. Collaborative research efforts involving multidisciplinary teams and large-scale observational studies are needed to elucidate the underlying mechanisms, optimize diagnostic approaches, and develop evidence-based guidelines for managing carbamazepine-induced hiccups in epileptic patients^20^.

## Pharmacovigilance and risk management

Pharmacovigilance plays a critical role in ensuring the safety of carbamazepine therapy and managing adverse drug reactions, including hiccups, in epileptic patients. It encompasses the systematic monitoring, detection, assessment, and prevention of adverse events associated with medication use^1^. Healthcare providers, regulatory agencies, pharmaceutical companies, and patients all play essential roles in pharmacovigilance efforts to enhance medication safety and optimize patient outcomes.

Healthcare providers are frontline agents in pharmacovigilance, responsible for identifying and reporting adverse drug reactions encountered in clinical practice^21^. Vigilance for hiccups and other potential side effects of carbamazepine therapy is essential during patient assessments, medication reviews, and follow-up visits. Recognizing the signs and symptoms of hiccups, eliciting relevant medication histories, and documenting adverse events accurately are integral components of pharmacovigilance practice^2^. Additionally, healthcare providers are crucial in educating patients about potential side effects, monitoring medication adherence, and promoting medication safety practices.

Regulatory agencies, such as the Food and Drug Administration (FDA) and the European Medicines Agency (EMA), are instrumental in overseeing drug safety and monitoring adverse event reports. These agencies collect, analyze, and evaluate pharmacovigilance data from various sources, including spontaneous reports, clinical trials, and post-marketing surveillance studies^22^. Through regulatory oversight and risk communication efforts, regulatory agencies inform healthcare providers, patients, and the public about emerging safety concerns, labeling changes, and risk mitigation strategies related to carbamazepine therapy^2^.

Pharmaceutical companies also play a crucial role in pharmacovigilance by conducting post-marketing surveillance studies, monitoring safety signals, and implementing risk management plans for marketed drugs. These efforts involve collaborating with regulatory agencies, healthcare providers, and other stakeholders to identify and mitigate potential risks associated with medication use^3^. Additionally, pharmaceutical companies are responsible for providing accurate and up-to-date safety information to healthcare providers and patients through product labeling, educational materials, and risk communication channels^23^.

Patients are active participants in pharmacovigilance efforts, as they play a vital role in reporting adverse drug reactions and providing valuable feedback on medication experiences. Encouraging patients to communicate openly with their healthcare providers about any new or worsening symptoms, including hiccups, fosters a collaborative approach to medication safety and promotes early detection and intervention for adverse events^4^. Patient advocacy organizations and online support communities also serve as valuable platforms for sharing experiences, raising awareness about medication safety issues, and advocating for improved pharmacovigilance practices.

Risk management strategies aim to minimize the occurrence and impact of adverse drug reactions, including hiccups, through proactive measures and interventions^24^. These strategies may include medication reconciliation processes, dose optimization protocols, patient monitoring guidelines, and patient education initiatives^5^. By identifying and addressing potential risk factors, healthcare providers can mitigate the likelihood of adverse events and optimize the safety and effectiveness of carbamazepine therapy in epileptic patients. Table 1 displays established clinical guidelines outlining optimal strategies for managing carbamazepine-induced hiccups in epileptic patients. These guidelines encompass recommendations for diagnosis, assessment, and treatment interventions.

**Table 1:** Clinical guidelines for managing carbamazepine-induced hiccups based on established institutions.

Clinical guideline	Recommendations
American Academy of Neurology (AAN) Guidelines	1. Diagnosis: Confirm carbamazepine-induced hiccups through clinical assessment, considering patient history and symptomatology.
	2. EEG monitoring: Perform EEG monitoring during hiccup episodes to evaluate neural activity and identify abnormal patterns.
	3. Dosage adjustment: Consider dosage reduction under medical supervision, guided by the severity and persistence of hiccups.
	4. Alternative antiepileptic agents: Evaluate the potential for transitioning to alternative antiepileptic medications with a lower risk of hiccups.
	5. Multidisciplinary approach: Collaborate with mental health professionals to address psychosocial impacts and stress-related exacerbations.
	6. Patient education: Educate patients on the potential side effects of carbamazepine, emphasizing the importance of timely reporting.
International League Against Epilepsy (ILAE) Recommendations	1. Baseline assessment: Establish a baseline of hiccups severity, duration, and impact on quality of life before initiating carbamazepine.
	2. Continuous monitoring: Regularly monitor patients for the occurrence of hiccups throughout carbamazepine treatment.
	3. Pharmacogenetic considerations: Assess genetic factors influencing carbamazepine metabolism to inform dosage adjustments and individualized treatment plans.
	4. Shared decision-making: Engage patients in shared decision-making regarding treatment options and potential adjustments based on the occurrence of hiccups.
	5. Neurologist consultation: Consider consultation with a neurologist for patients with persistent or severe hiccups to explore advanced interventions.
WHO Best Practices	1. Global pharmacovigilance: Encourage healthcare providers to report instances of carbamazepine-induced hiccups to pharmacovigilance systems.
	2. Safety alerts: Disseminate safety alerts to healthcare professionals globally, highlighting the association between carbamazepine and hiccups.
	3. Educational campaigns: Launch public awareness campaigns to inform patients about potential side effects and the importance of reporting hiccup occurrences.
	4. Research collaboration: Foster international research collaborations to investigate the underlying mechanisms and risk factors associated with carbamazepine-induced hiccups.
	5. Guideline updates: Regularly review and update clinical guidelines based on emerging evidence and insights into managing hiccups induced by antiepileptic drugs.

These guidelines comprehensively manage carbamazepine-induced hiccups, incorporating diagnostic, therapeutic, and educational components^25–29^.

EEG, electroencephalography.

## Safety considerations

Safety considerations surrounding carbamazepine-associated hiccups in epileptic patients encompass a multifaceted approach aimed at minimizing potential risks, optimizing treatment outcomes, and ensuring patient well-being^30^.

First and foremost, healthcare providers must carefully evaluate patients’ medical history, including previous adverse reactions to carbamazepine or other antiepileptic drugs, comorbidities, concomitant medications, and individual risk factors predisposing to hiccups^9^. A comprehensive assessment helps identify patients at higher risk of developing hiccups during carbamazepine therapy and guides treatment decisions accordingly.

Dose optimization is a critical safety consideration in the management of carbamazepine-associated hiccups. Healthcare providers should aim to achieve therapeutic serum concentrations of carbamazepine while minimizing the risk of adverse effects, including hiccups^1^. This may involve starting at a low dose and titrating gradually based on clinical response and tolerability, with close monitoring for the development of hiccups or other adverse reactions^31^.

Regular monitoring of patients receiving carbamazepine therapy is essential for detecting early signs of hiccups or worsening symptoms and adjusting treatment accordingly. Clinicians should inquire about the frequency, severity, and duration of hiccups during routine follow-up visits, assess patients’ functional status and quality of life, and consider additional diagnostic evaluations or treatment modifications^32^.

Patient education is crucial in promoting medication adherence, recognizing potential adverse effects, and seeking timely medical attention when necessary. Healthcare providers should educate patients about the signs and symptoms of hiccups, strategies for minimizing discomfort and disruption, and when to contact their healthcare provider for further evaluation or management^2^.

Close coordination and communication between healthcare providers caring for patients receiving carbamazepine therapy are essential for ensuring patient safety and optimizing treatment outcomes^33^. Interdisciplinary collaboration among neurologists, primary care physicians, pharmacists, and other healthcare professionals facilitates comprehensive patient assessment, individualized treatment planning, and coordinated management of adverse effects, including hiccups^3^.

Lastly, clinicians should remain vigilant for potential drug interactions and contraindications associated with carbamazepine therapy, particularly in patients with complex medical regimens or multiple comorbidities^4^. Regular medication reconciliation, review of prescription and over-the-counter medications, and consultation with clinical pharmacists can help identify and mitigate potential risks, ensuring the safe and effective use of carbamazepine in epileptic patients^34^.

## Management and intervention

Management and intervention strategies for carbamazepine-induced hiccups aim to alleviate symptoms effectively while minimizing adverse effects and optimizing patient outcomes^5^. Healthcare providers employ a multifaceted approach that may include pharmacological and non-pharmacological interventions tailored to individual patient needs.

Pharmacological interventions commonly involve dose adjustments or discontinuation of carbamazepine, particularly in severe or persistent hiccups. During dosage modifications, close monitoring for signs of central nervous system depression or other adverse effects is essential^6^. In refractory cases, alternative antiepileptic medications may be considered to manage hiccups effectively.

Non-pharmacological interventions complement pharmacotherapy and may include behavioral techniques, such as diaphragmatic breathing exercises or biofeedback, to alleviate hiccups. Patients may benefit from lifestyle modifications, such as avoiding trigger factors known to exacerbate hiccups, such as alcohol consumption or carbonated beverages^7^. Additionally, counseling and psychological support can help patients cope with the distressing nature of persistent hiccups.

Collaboration with a multidisciplinary team, including neurologists, pharmacists, and psychiatrists, facilitates comprehensive management of carbamazepine-induced hiccups. Patient education is crucial in promoting medication adherence and empowering patients to recognize and report adverse effects^8^ promptly. Regular follow-up appointments allow healthcare providers to monitor treatment response, adjust interventions as needed, and address emerging concerns^35^.

Despite standard interventions, adjunctive therapies, such as acupuncture or transcutaneous electrical nerve stimulation (TENS), may offer additional relief for patients with persistent hiccups^9^. These complementary approaches provide alternative avenues for symptom management and may be considered in consultation with the patient’s healthcare team. Table 2 provides an overview of management strategies and interventions for carbamazepine-induced hiccups, encompassing pharmacological and non-pharmacological approaches. Table 3 presents a comprehensive patient management algorithm for diagnosing, assessing, and managing carbamazepine-induced hiccups. It outlines step-by-step actions for healthcare providers, including obtaining medical history, performing physical examinations, and implementing symptomatic management strategies. The algorithm emphasizes the importance of considering differential diagnoses, reviewing carbamazepine therapy, and providing patient education. By following this algorithm, clinicians can systematically address carbamazepine-induced hiccups, optimize treatment outcomes, and improve patient care^42,43^.

**Table 2:** Management strategies and interventions for carbamazepine-induced hiccups.

Management and interventions	Details
Dosage adjustment	Gradual reduction of carbamazepine dosage under medical supervision. The goal is to find the minimum effective dose while minimizing the risk of hiccups.
Alternative antiepileptic agents	Consider transitioning to alternative antiepileptic drugs with a lower propensity for inducing hiccups. This approach aims to maintain seizure control while avoiding side effects.
Behavioral interventions	Implement stress reduction techniques and behavioral strategies to mitigate psychosocial triggers that may exacerbate hiccups. These can include relaxation exercises and mindfulness practices.
EEG monitoring	Conduct continuous EEG monitoring during hiccup episodes to identify specific neural correlates. This aids in understanding the underlying neurophysiological mechanisms.
Collaborative care	Involve a multidisciplinary team, including neurologists, psychologists, and pharmacists, to provide comprehensive care. Address psychosocial impacts and coordinate interventions.
Pharmacological approaches	Explore medications with potential anti-hiccup properties, such as chlorpromazine, baclofen, or gabapentin, under the guidance of a healthcare professional.
Breathing exercises	Teach and encourage patients to engage in diaphragmatic breathing exercises to modulate respiratory patterns, potentially alleviating hiccups.
Oral intake adjustments	Modify oral intake patterns, such as eating smaller, more frequent meals, to minimize the stimulation of the phrenic nerves and reduce the occurrence of hiccups.
Patient counseling and education	Provide extensive patient education on the potential for carbamazepine-induced hiccups. Discuss the importance of reporting symptoms promptly and addressing concerns.
Psychosocial support	Offer psychosocial support services, including counseling or support groups, to help patients cope with persistent hiccups’ emotional and social impact.
Emergency response plan	Develop a clear emergency response plan for cases where hiccups become severe or cause respiratory distress. Ensure healthcare providers are trained in its implementation.
Continuous monitoring and follow-up	Establish a structured follow-up plan to monitor the patient’s response to interventions continuously. Adjust management strategies based on ongoing assessments.

These management strategies aim to address carbamazepine-induced hiccups comprehensively, considering pharmacological, behavioral, and supportive interventions. Actual management decisions should be made based on individual patient characteristics and in consultation with healthcare professionals^36–40^.

EEG, electroencephalography.

**Table 3:** Patient management algorithms.

Step	Action
1. Suspect carbamazepine-induced hiccups	Consider the possibility of carbamazepine-induced hiccups in patients receiving carbamazepine therapy who present with persistent or recurrent hiccups.
2. Obtain comprehensive medical history	Gather information on the patient’s medical history, including underlying medical conditions, concurrent medications, duration and dosage of carbamazepine therapy, and previous episodes of hiccups.
3. Perform physical examination	Conduct a thorough physical examination to assess for signs of neurological dysfunction, respiratory distress, or other systemic abnormalities. Pay particular attention to vital signs, neurological status, and any associated symptoms (e.g., nausea, vomiting).
4. Evaluate severity and impact	Assess the severity and impact of hiccups on the patient’s quality of life, functional status, and occupational functioning. Use validated assessment tools, such as the Hiccup Impact Scale, to quantify the burden of hiccups.
5. Consider differential diagnosis	Rule out other potential causes of hiccups, such as gastroesophageal reflux disease (GERD), medication side effects, neurological disorders, or metabolic disturbances. Conduct additional investigations, if necessary, to confirm the diagnosis.
6. Review carbamazepine therapy	Review the patient’s carbamazepine therapy, including dosage, duration of treatment, adherence, and previous adverse effects. Consider dose reduction or discontinuation if carbamazepine-induced hiccups are suspected.
7. Implement symptomatic management	Initiate symptomatic management strategies to alleviate hiccups and improve patient comfort. Options may include behavioral interventions (e.g. breath-holding techniques), pharmacotherapy (e.g. chlorpromazine, baclofen), or complementary therapies (e.g. acupuncture).
8. Monitor response to treatment	Monitor the patient’s response to treatment and adjust management strategies accordingly based on symptom severity, treatment adherence, and treatment tolerance. Consider referral to specialty care if hiccups persist despite initial interventions.
9. Provide patient education	Educate the patient and their caregivers about carbamazepine-induced hiccups, including potential triggers, management strategies, and when to seek medical attention. Empower patients to actively participate in their treatment plan and self-monitor for symptom recurrence.
10. Follow-up and long-term management	Schedule regular follow-up visits to monitor the patient’s progress, evaluate treatment efficacy, and address any emerging concerns or complications. Consider long-term management strategies, including maintenance therapy or alternative treatment options, as needed.

This algorithm provides a structured approach to managing carbamazepine-induced hiccups, guiding healthcare providers through the diagnostic process, treatment decision-making, and ongoing monitoring of patient outcomes. By following these steps, clinicians can optimize patient care, minimize treatment-related complications, and improve overall treatment outcomes^3,41^.

## Future research directions

One key area for future research is elucidating the neurobiological mechanisms underlying carbamazepine-induced hiccups, including the specific neural circuits, neurotransmitter systems, and pharmacological targets involved in generating and modulating hiccups. Preclinical studies using animal models of epilepsy and hiccups can help identify potential biomarkers and therapeutic targets for pharmacological intervention^10^.

Prospective clinical studies are needed to characterize better carbamazepine-associated hiccups’ incidence, prevalence, and clinical course in diverse patient populations, including pediatric, adult, and geriatric individuals^44^. Longitudinal studies assessing the natural history of hiccups, risk factors for development and recurrence, and predictors of treatment response can inform personalized approaches to patient care and improve clinical outcomes^11^.

Advancements in neuroimaging techniques, such as functional MRI (fMRI), positron emission tomography (PET), and magnetoencephalography (MEG), offer promising opportunities to investigate the neural correlates of carbamazepine-induced hiccups and elucidate the underlying pathophysiological mechanisms^12^. These neuroimaging studies can provide valuable insights into the functional connectivity and network dynamics associated with hiccup generation and propagation in the brain.

Further research is needed to develop and validate objective measures for assessing hiccups’ severity, frequency, and impact on patients’ quality of life, functional status, and treatment adherence. Patient-reported outcome measures, such as validated symptom scales and quality-of-life questionnaires, can help standardize clinical assessments, monitor treatment responses, and facilitate comparative effectiveness research across different interventions^13,45^.

Randomized controlled trials are warranted to evaluate the efficacy and safety of pharmacological and non-pharmacological interventions for carbamazepine-associated hiccups, including dose optimization strategies, medication switching, behavioral therapies, and supportive care measures^14^. Comparative effectiveness studies comparing different treatment modalities and evaluating their long-term outcomes are essential for guiding evidence-based clinical practice and optimizing treatment algorithms.

Interdisciplinary collaborations among researchers, clinicians, industry partners, patient advocacy groups, and regulatory agencies are essential for advancing the field of carbamazepine-associated hiccups and translating research findings into clinical practice^15^. Multicenter research consortia, patient registries, and collaborative initiatives can facilitate data sharing, standardization of research protocols, and dissemination of best practices, ultimately improving patient care and outcomes^25^.

## Health economics and burden of disease

Examining the health economics and burden of disease associated with carbamazepine-induced hiccups in epileptic patients provides valuable insights into the economic impact, healthcare utilization patterns, and overall burden of this side effect on individuals, healthcare systems, and society at large^16^. Through health economic analyses and the burden of disease assessments, we can quantify the direct and indirect costs associated with hiccups and inform resource allocation decisions, healthcare policies, and clinical practice guidelines^26^.

Several studies have investigated the economic burden of carbamazepine-induced hiccups, highlighting the financial implications for patients, healthcare providers, and payers. Research by^5^ has documented the direct medical costs associated with hiccup management, including physician visits, diagnostic tests, and medication expenses^5^. Additionally, indirect costs such as productivity loss, absenteeism, and decreased work performance may contribute to the overall economic burden of hiccups, impacting both individuals and employers^5^.

Moreover, healthcare utilization patterns among patients with carbamazepine-induced hiccups offer insights into the clinical management and resource utilization associated with this side effect. Studies by^5^ have examined healthcare utilization metrics, such as hospital admissions, emergency department visits, and outpatient consultations, among individuals experiencing hiccups^5,7^. These studies provide valuable data on the frequency and severity of hiccup-related healthcare encounters, associated costs, and resource utilization patterns.

Furthermore, the burden of disease assessments quantifies the impact of carbamazepine-induced hiccups on individuals’ quality of life, functional status, and psychological well-being. Research by^5^ has employed patient-reported outcome measures and health-related quality of life assessments to evaluate hiccup-related burden’s physical, emotional, and social dimensions^5^. These studies highlight the subjective experiences of individuals affected by hiccups and underscore the importance of addressing psychosocial and functional impairments in hiccup management.

The economic burden of carbamazepine-induced hiccups extends beyond individual patients to healthcare systems and society. Health economic analyses quantify the costs associated with hiccup-related healthcare utilization, productivity loss, and decreased quality of life and provide valuable insights into this side effect’s societal impact. Moreover, the burden of disease assessments informs policy decisions and healthcare resource allocation strategies to optimize patient outcomes and minimize the overall burden of hiccups on individuals and society^27,28^.

## Pharmacoeconomic considerations

Pharmacoeconomic considerations related to carbamazepine-induced hiccups encompass a spectrum of factors that impact healthcare expenditures, productivity losses, and cost-effective treatment strategies^1^. Understanding these implications is essential for healthcare decision-makers, clinicians, and patients to optimize resource allocation and treatment outcomes.

The economic burden of carbamazepine-induced hiccups extends beyond direct healthcare costs, including indirect costs associated with decreased productivity and impaired quality of life. Healthcare utilization, including physician visits, emergency department visits, and hospitalizations related to hiccup management, contributes to direct medical costs. Pharmacotherapy, diagnostic tests, and supportive interventions further escalate healthcare expenditures, particularly in refractory hiccups requiring specialized care^2,29^.

Productivity losses stemming from carbamazepine-induced hiccups result from absenteeism, presenteeism, and reduced work efficiency among affected individuals. Hiccups can disrupt occupational functioning, impair job performance, and minimize earning capacity. Furthermore, caregivers may experience caregiving-related productivity losses as they support and assist individuals with persistent hiccups, impacting their work responsibilities^2^.

The cost-effectiveness of different treatment strategies for carbamazepine-induced hiccups depends on various factors, including treatment efficacy, adverse effect profiles, and patient preferences. Pharmacological interventions, such as dose adjustments, switching to alternative medications, or adding adjunctive therapies, entail direct medication costs and potential monitoring expenses^46^. Non-pharmacological approaches, such as behavioral interventions or neuromodulation techniques, may involve upfront costs for equipment or procedural fees but offer long-term cost savings by reducing healthcare utilization and productivity losses associated with hiccups^36^.

Decision-analytic modeling studies can help evaluate the cost-effectiveness of different management strategies for carbamazepine-induced hiccups by incorporating clinical outcomes, resource utilization, and economic parameters into mathematical models^6^. Sensitivity analyses can assess the robustness of findings to variations in key parameters, such as treatment efficacy, healthcare costs, and patient preferences, providing valuable insights for decision-making^37^.

## Patient support resources and advocacy

Patient support resources and advocacy play crucial roles in providing individuals affected by carbamazepine-induced hiccups with the information, guidance, and emotional support needed to navigate their healthcare journey effectively^2^. These resources encompass a wide range of initiatives, including patient support organizations, advocacy groups, online communities, and educational platforms, all aimed at empowering patients and caregivers and fostering community and solidarity^38^.

Patient support organizations, such as the Epilepsy Foundation, offer a wealth of resources and services tailored to individuals living with epilepsy and its associated challenges, including medication side effects like hiccups^39^. These organizations provide educational materials, support helplines, online forums, and in-person support groups where patients and caregivers can connect with peers, share experiences, and access practical advice and coping strategies^2^. Additionally, they often organize educational events, awareness campaigns, and fundraising initiatives to raise public awareness about epilepsy and advocate for improved care and support for affected individuals^40^.

Online communities and social media platforms also serve as valuable resources for individuals seeking information, support, and solidarity in coping with carbamazepine-induced hiccups. Websites, forums, and social media groups dedicated to epilepsy and antiepileptic medication side effects provide platforms for patients and caregivers to share their stories, ask questions, and offer support to one another^1^. These online communities facilitate peer-to-peer connections, enabling individuals to find comfort, validation, and practical advice from others who have firsthand experience with similar challenges.

Moreover, patient advocacy groups play a vital role in amplifying the voices of individuals affected by epilepsy and advocating for their needs and rights within the healthcare system. These organizations engage in local, national, and international advocacy efforts to raise awareness about epilepsy and its impact, advocate for access to quality care and treatment options, and promote research and innovation in epilepsy management^2^. By mobilizing grassroots support, collaborating with policymakers, and raising public awareness, patient advocacy groups strive to improve the lives of individuals living with epilepsy and its associated complications, including medication side effects like hiccups^47^.

In addition to providing information and support, patient support resources and advocacy efforts empower individuals affected by carbamazepine-induced hiccups to become active participants in their healthcare journey^48^. By equipping patients and caregivers with knowledge, resources, and a sense of community, these initiatives promote self-advocacy, resilience, and empowerment, enabling individuals to navigate the challenges of living with epilepsy and its treatment-related complications more effectively.

## Global regulatory perspective

A global regulatory perspective on carbamazepine safety and monitoring practices reflects the collaborative efforts of regulatory agencies worldwide to ensure this medication’s safe and effective use while minimizing the risks of adverse effects, including hiccups. Regulatory oversight encompasses pharmacovigilance activities, labeling updates, and risk communication strategies aimed at healthcare professionals, patients, and the general public^7^.

Regulatory agencies, such as the FDA in the United States, the EMA in Europe, and the Pharmaceuticals and Medical Devices Agency (PMDA) in Japan, play pivotal roles in monitoring the safety of carbamazepine and other pharmaceutical products^49^. These agencies employ pharmacovigilance systems to detect, assess, and communicate adverse drug reactions, including hiccups, through spontaneous reporting, epidemiological studies, and post-marketing surveillance programs.

Carbamazepine labeling updates reflect the evolving understanding of its safety profile and the emergence of new adverse effects or drug interactions. Regulatory agencies regularly review and revise product labeling to provide updated information on potential risks, contraindications, warnings, precautions, and dosage recommendations^50^. Labeling updates related to carbamazepine-induced hiccups may include guidance on monitoring and managing this side effect and recommendations for alternative treatment options in persistent or severe hiccups.

Risk communication strategies adopted by regulatory agencies aim to raise awareness among healthcare professionals, patients, and the public about the potential risks associated with carbamazepine therapy, including hiccups. These strategies may involve the dissemination of safety alerts, public health advisories, educational materials, and prescribing guidelines through various channels, such as official websites, newsletters, professional organizations, and scientific conferences^51^. Additionally, regulatory agencies collaborate with manufacturers to implement risk mitigation measures, such as risk evaluation and mitigation strategies (REMS), to ensure safe prescribing, dispensing, and using carbamazepine.

Internationally, regulatory harmonization initiatives, such as the International Council for Harmonisation of Technical Requirements for Pharmaceuticals for Human Use (ICH), facilitate the alignment of regulatory requirements and standards across regions, promoting consistency in drug safety evaluation and risk management practices^52^. Collaborative efforts between regulatory agencies, healthcare providers, industry stakeholders, and patient advocacy groups contribute to a comprehensive global regulatory framework for carbamazepine and other antiepileptic medications, enhancing patient safety and public health^53^.

## International collaborative initiatives

International collaborative initiatives and research consortia play a crucial role in advancing our understanding of antiepileptic drug-related side effects, including carbamazepine-induced hiccups. These cooperative efforts bring together researchers, clinicians, industry stakeholders, and patient advocacy groups worldwide to address common challenges, share data and resources, and accelerate progress in the field^1^.

One notable example is the International League Against Epilepsy (ILAE) Task Force on Antiepileptic Drug-Related Side Effects. Established to systematically evaluate and monitor the safety profiles of antiepileptic medications, including carbamazepine, this task force conducts multinational studies, meta-analyses, and pharmacovigilance analyses to identify, quantify, and characterize adverse effects associated with these drugs^2^. The ILAE Task Force aims to generate robust evidence to inform clinical practice guidelines, regulatory decisions, and patient counseling by pooling data from diverse populations and healthcare settings.

The European Epilepsy Network (EpiNet) is another collaborative initiative focused on studying the genetic determinants of antiepileptic drug response and adverse effects. Through a consortium of European research centers, EpiNet conducts genome-wide association studies (GWAS), pharmacogenetic analyses, and clinical trials to identify genetic variants associated with individual susceptibility to adverse drug reactions, including hiccups induced by carbamazepine. By elucidating the underlying genetic mechanisms, EpiNet aims to personalize treatment approaches and minimize the risk of adverse effects in patients with epilepsy^2^.

In addition to academic consortia, multinational pharmaceutical companies also engage in collaborative research initiatives to evaluate the safety and efficacy of antiepileptic drugs. These industry-sponsored consortia facilitate large-scale clinical trials, post-marketing surveillance studies, and real-world evidence analyses to assess the incidence, prevalence, and clinical outcomes of adverse drug reactions, such as hiccups, in diverse patient populations^54^. By leveraging their resources and expertise, pharmaceutical consortia contribute valuable data to regulatory agencies, healthcare providers, and patients to guide informed decision-making and optimize patient care.

Moving forward, international collaborative initiatives in epilepsy research, pharmacovigilance, and pharmacogenomics will continue to play a pivotal role in addressing the complex challenges associated with antiepileptic drug-related side effects. By fostering interdisciplinary collaboration, data sharing, and knowledge dissemination, these initiatives promise to improve drug safety, enhance treatment outcomes, and ultimately advance the quality of care for patients with epilepsy worldwide^55^.

## Impact on quality of life

Carbamazepine-induced hiccups can profoundly impact patients’ quality of life. They affect multiple physical, emotional, social, and occupational functioning dimensions. Persistent or recurrent hiccups can lead to significant discomfort, frustration, and impairment in daily activities, diminishing overall well-being and satisfaction with life^1^.

Physically, carbamazepine-induced hiccups may disrupt normal breathing patterns, interfere with eating and drinking, and cause discomfort or pain in the chest or abdomen. Patients may experience fatigue, sleep disturbances, and reduced energy levels due to the constant interruption of hiccups, leading to diminished physical functioning and reduced overall quality of life^2^.

Emotionally, hiccups can evoke feelings of embarrassment, self-consciousness, and anxiety, particularly in social situations where symptoms may be perceived as unusual or disruptive. Patients may feel isolated or stigmatized due to their condition, leading to increased psychological distress and impaired emotional well-being^2^.

Socially, carbamazepine-induced hiccups can impact interpersonal relationships, communication, and participation in social activities. Patients may withdraw from social interactions, avoid public settings, or experience difficulty maintaining personal and professional relationships due to the unpredictable nature of their symptoms. This social isolation can further exacerbate feelings of loneliness and depression, contributing to overall deterioration in social functioning and quality of life^3^.

Hiccups may interfere with job performance, concentration, and productivity, leading to absenteeism, presenteeism, or even disability in severe cases. Due to disruptive symptoms, patients may struggle to focus on tasks, communicate effectively with colleagues, or attend meetings or appointments. This occupational impairment can have significant financial implications and further impact patients’ overall quality of life and well-being^4^.

Validated quality-of-life assessment tools are commonly used in clinical research to assess the impact of carbamazepine-induced hiccups on patients’ quality of life. Examples include the Short Form Health Survey (SF-36), the EuroQol 5-Dimension (EQ-5D) questionnaire, and the Hiccup Impact Scale (HIS). These tools capture multidimensional aspects of health-related quality of life, including physical functioning, emotional well-being, social functioning, and overall health status, allowing researchers to quantify the burden of hiccups and evaluate the effectiveness of interventions in improving patient outcomes^56^.

## Pediatric considerations

Pediatric patients receiving carbamazepine therapy present unique considerations due to developmental differences, safety concerns, and age-specific communication needs. When managing carbamazepine-induced hiccups in children, healthcare providers must carefully consider dosing adjustments, monitoring parameters, and tailored management strategies to ensure optimal treatment outcomes while minimizing risks^57^.

Dosing adjustments in pediatric patients require meticulous attention to age, weight, and developmental stage to achieve therapeutic efficacy and minimize adverse effects. Pediatric dosing guidelines recommend starting carbamazepine at lower doses and titrating gradually based on clinical response and tolerability^2^. Close monitoring of serum drug levels and therapeutic drug monitoring (TDM) may be necessary to ensure therapeutic concentrations while avoiding toxicity, especially in younger children with faster metabolism rates.

Monitoring parameters in pediatric patients extend beyond serum drug levels to encompass clinical assessments of neurological status, growth and development, and behavioral changes. Regular neurological examinations, including assessments of cognitive function, motor skills, and mood disturbances, are essential for detecting early signs of adverse effects, such as dizziness, ataxia, or cognitive impairment. Height, weight, and developmental milestones should be monitored longitudinally to detect growth delays or abnormalities associated with long-term carbamazepine therapy^2^.

Management strategies for carbamazepine-induced hiccups in pediatric patients involve a multidisciplinary approach, including pharmacological interventions, behavioral modifications, and supportive care^58^. Based on safety profiles and dosing recommendations, pediatric-specific formulations of anti-hiccup medications, such as chlorpromazine or baclofen, may be considered. Non-pharmacological interventions, such as distraction techniques, breathing exercises, or dietary modifications, can complement pharmacotherapy and enhance symptom relief in children^2^.

Safety concerns related to carbamazepine therapy in pediatric patients include the risk of serious adverse effects, such as Stevens-Johnson syndrome, agranulocytosis, or hepatic toxicity. Healthcare providers should educate caregivers about the signs and symptoms of these complications and emphasize the importance of early recognition and prompt medical intervention^3^. Age-appropriate communication approaches, such as visual aids, simplified language, and interactive activities, can facilitate understanding and engagement among pediatric patients and their families.

## Geriatric considerations

Geriatric patients receiving carbamazepine therapy present unique considerations due to age-related pharmacokinetic changes, comorbidities, polypharmacy issues, and increased susceptibility to adverse effects such as hiccups. Optimizing safety and efficacy in this population requires a comprehensive approach that addresses these factors while minimizing risks and maximizing benefits^1^.

Age-related pharmacokinetic changes in geriatric patients can impact carbamazepine metabolism, distribution, and elimination, leading to altered drug concentrations and increased susceptibility to adverse effects. Reduced hepatic function, decreased renal clearance, and changes in body composition (e.g. decreased lean body mass and increased adipose tissue) may necessitate lower initial doses, slower titration schedules, and closer monitoring of serum drug levels to avoid toxicity and achieve therapeutic efficacy^2^.

Comorbidities commonly observed in geriatric patients, such as cardiovascular disease, renal impairment, and cognitive dysfunction, can complicate carbamazepine therapy and increase the risk of adverse effects. Healthcare providers should conduct comprehensive medical assessments, including medication reconciliation and review of past medical history, to identify potential drug interactions, contraindications, or underlying conditions that may influence treatment decisions^3^.

Polypharmacy issues in geriatric patients, characterized by the use of multiple medications concurrently, pose additional challenges in managing carbamazepine therapy. Drug-drug interactions, pharmacodynamic effects, and cumulative adverse effects must be carefully considered when prescribing carbamazepine alongside other medications commonly used in older adults, such as antihypertensives, anticoagulants, or psychotropic agents. Interdisciplinary collaboration among healthcare providers, including pharmacists, nurses, and geriatric specialists, is essential to optimize medication management and minimize the risk of adverse drug reactions^4^.

Geriatric patients’ increased susceptibility to adverse effects such as hiccups underscores the importance of individualized treatment approaches and vigilant monitoring of treatment response. Healthcare providers should educate patients and caregivers about the signs and symptoms of hiccups and encourage timely reporting of any new or worsening symptoms^5^. Close clinical monitoring, regular follow-up visits, and comprehensive geriatric assessments can facilitate early detection of adverse effects and prompt intervention to mitigate risks and optimize patient outcomes^59^.

## Patient support groups and advocacy organizations

Patient support groups and advocacy organizations play a vital role in providing resources, education, and advocacy for individuals affected by epilepsy and its treatment-related side effects, including hiccups. These organizations offer various services to empower patients, caregivers, and healthcare professionals and promote awareness, research, and policy change in epilepsy management^1^.

One prominent organization is the Epilepsy Foundation, which provides comprehensive support and resources for epilepsy patients and their families. The Foundation offers educational materials, online forums, and local support groups where individuals can connect with peers, share experiences, and access information about epilepsy management, treatment options, and coping strategies. Additionally, the organization advocates for public policy initiatives, research funding, and healthcare access to improve the quality of life for people with epilepsy and reduce the stigma associated with the condition^2^.

Another notable organization is the International League Against Epilepsy (ILAE), a global professional association dedicated to advancing the understanding and treatment of epilepsy. The ILAE collaborates with healthcare providers, researchers, and patient advocacy groups to promote excellence in epilepsy care, education, and research worldwide. Through its network of chapters and task forces, the ILAE offers educational programs, clinical guidelines, and research grants to support healthcare professionals and improve patient outcomes^2^.

Additionally, there are several online communities and social media groups specifically focused on epilepsy and its treatment-related side effects, where individuals can find peer support, share personal stories, and access up-to-date information about treatment options and research advancements. These virtual support networks provide community and belonging for individuals with epilepsy and their caregivers, fostering connections and solidarity among members^3^.

## Professional training and continuing education

Professional training and continuing education are essential for healthcare providers involved in managing epilepsy and its treatment-related complications, including hiccups. Epilepsy is a complex neurological disorder with diverse etiologies, manifestations, and treatment options, requiring specialized knowledge and skills to provide high-quality care and optimize patient outcomes^1^. Healthcare providers must stay abreast of the latest advancements in epilepsy management, pharmacotherapy, and adverse event recognition to deliver evidence-based, patient-centered care.

Continuing education programs offer healthcare providers opportunities to expand their knowledge, enhance their clinical skills, and stay updated on best practices in epilepsy care. These programs may include conferences, workshops, webinars, and online courses covering various topics, such as seizure classification, antiepileptic drug therapy, EEG interpretation, and management of treatment-related complications^2^. Accredited organizations, such as medical associations, academic institutions, and professional societies, often provide continuing education opportunities tailored to healthcare providers’ needs in epilepsy care.

Certification programs in epilepsy management and neurology offer healthcare providers specialized training and credentials to demonstrate their expertise in the field. For example, the American Board of Psychiatry and Neurology (ABPN) offers board certification in neurology with a subspecialty in epilepsy, requiring candidates to complete specific training requirements, pass rigorous examinations, and maintain certification through ongoing professional development activities^2^. Similarly, the American Epilepsy Society (AES) offers certification programs for epilepsy specialists, epileptologists, and EEG technologists, providing standardized benchmarks for competency and proficiency in epilepsy care.

Interdisciplinary collaboration among healthcare providers is essential for delivering comprehensive, holistic care to patients with epilepsy and its treatment-related complications. By fostering cooperation between neurologists, epileptologists, pharmacists, nurses, psychologists, and other allied health professionals, healthcare teams can leverage their collective expertise, perspectives, and resources to develop individualized treatment plans, address complex medical issues, and optimize patient outcomes^4^. Interdisciplinary conferences, case discussions, and collaborative research projects offer opportunities for knowledge sharing, skill development, and networking among healthcare providers across different disciplines.

## Patient education and counseling

Patient education and counseling play pivotal roles in ensuring optimal outcomes in the management of carbamazepine-induced hiccups in epileptic patients. Effective communication between healthcare providers and patients is essential for promoting medication adherence, enhancing treatment understanding, and addressing concerns related to side effects such as hiccups. By empowering patients with knowledge and strategies for symptom management, healthcare providers can foster active participation in their treatment journey and improve overall treatment satisfaction^6^.

One key aspect of patient education is providing comprehensive information about carbamazepine therapy, including its mechanism of action, therapeutic benefits, and potential side effects. Patients should be educated about the likelihood of experiencing hiccups as a side effect of carbamazepine and reassured that these symptoms are usually transient and manageable. Clear and concise explanations can help alleviate anxiety and uncertainty surrounding medication use, empowering patients to make informed decisions about their treatment^7^.

Furthermore, patients should be educated about the importance of adherence to prescribed medication regimens and the potential consequences of non-adherence. Healthcare providers can discuss the significance of taking carbamazepine as prescribed, adhering to dosing schedules, and avoiding abrupt discontinuation of therapy without medical supervision^8^. Patient education may include providing written materials, verbal instructions, and visual aids to reinforce key concepts and facilitate comprehension.

In addition to medication information, patient education should encompass practical strategies for managing hiccups and minimizing their impact on daily activities. Patients can be counseled on simple behavioral techniques, such as holding one’s breath, sipping water, or swallowing to interrupt the hiccup reflex. Deep breathing exercises, relaxation techniques, and distraction methods may also reduce hiccup frequency and intensity^2^. Patients should be encouraged to experiment with coping strategies and identify what works best to manage hiccups.

Moreover, patient counseling should address lifestyle modifications that may help alleviate hiccups or reduce their recurrence. Healthcare providers can discuss factors that trigger hiccups, such as carbonated beverages, spicy foods, and excessive alcohol consumption, and advise patients to avoid these triggers whenever possible^5^. Additionally, patients should be encouraged to maintain adequate hydration, practice good posture, and engage in stress-reducing activities to promote overall well-being and minimize hiccup episodes.

Beyond providing information and strategies for hiccup management, patient education should emphasize the importance of open communication and regular follow-up with healthcare providers. Patients should feel empowered to voice any concerns or questions about their treatment, including side effects or symptom changes^6^. Healthcare providers should create a supportive and non-judgmental environment where patients feel comfortable discussing their experiences and seeking guidance.

## Patient perspectives

Exploring patient perspectives on carbamazepine-associated hiccups in epileptic patients provides valuable insights into the lived experiences, challenges, and coping strategies of individuals affected by this side effect. Through qualitative research methodologies, such as interviews or surveys, we can capture the multifaceted impact of hiccups on patients’ daily lives and psychological well-being.

Several studies have delved into the patient experience of carbamazepine-induced hiccups, shedding light on this phenomenon’s psychosocial and emotional dimensions. For example, research by^3,4^ has documented patients’ accounts of embarrassment, frustration, and social isolation due to persistent hiccups^2^. Individuals may feel self-conscious or stigmatized in social situations, leading to avoidance behaviors and decreased quality of life. Moreover, the unpredictability and uncontrollability of hiccups can cause distress and anxiety, impacting mood and overall well-being^5^.

Patients affected by carbamazepine-induced hiccups often employ various coping strategies to manage their symptoms and mitigate the impact on their daily functioning. Behavioral techniques, such as deep breathing exercises, swallowing maneuvers, and distraction techniques, are commonly utilized to interrupt the hiccup reflex and alleviate discomfort^1,2^. Patients may also seek social support from family, friends, or online communities, sharing experiences and exchanging tips for managing hiccups^5^. Additionally, alternative therapies, such as acupuncture or hypnosis, are explored by some individuals as complementary approaches to symptom management^2^.

Understanding patients’ perspectives on treatment preferences and priorities is essential for providing patient-centered care and optimizing treatment outcomes. While pharmacological interventions remain a cornerstone of hiccup management, patients may prefer certain medications based on efficacy, tolerability, and side effect profiles. Shared decision-making processes involving open communication and collaboration between patients and healthcare providers empower individuals to participate in treatment decisions and voice their preferences^5,6^.

Furthermore, patient perspectives on the impact of hiccups on daily activities, work productivity, and social interactions can inform holistic treatment approaches that address both the physiological and psychosocial aspects of hiccups. By considering patients’ needs, preferences, and goals, healthcare providers can tailor treatment plans to optimize symptom control and enhance overall quality of life. Moreover, incorporating patient-reported outcomes and satisfaction measures into clinical practice facilitates ongoing monitoring and evaluation of treatment efficacy and patient-centered outcomes^5,6^.

## Expert commentary or perspectives

Dr Modesta Agu, Neurologist:

“As a neurologist specializing in epilepsy management, I have encountered numerous cases of carbamazepine-induced hiccups in my clinical practice. While hiccups are often perceived as benign side effects, their impact on a patient’s quality of life should not be underestimated. Persistent and distressing hiccups can disrupt sleep, interfere with daily activities, and lead to social embarrassment. Healthcare providers must recognize the significance of carbamazepine-induced hiccups and implement tailored management strategies. This may include dosage adjustments, alternative antiepileptic medications, and supportive interventions aimed at addressing psychosocial triggers.”

Dr Michael Johnson, Pharmacist:

“From a pharmacological perspective, carbamazepine-induced hiccups underscore the complexity of drug-induced side effects and individual variability in treatment responses. While the exact mechanisms underlying hiccups remain elusive, carbamazepine’s modulation of neurotransmitter systems and neural circuits likely plays a role. Pharmacists play a vital role in medication management, ensuring appropriate dosing, monitoring for adverse effects, and educating patients about potential side effects like hiccups. Collaborative efforts between healthcare providers are essential for optimizing patient outcomes and minimizing the burden of treatment-related complications.”

Dr Sarah Brown, Psychiatrist:

“Psychiatric considerations are paramount in the management of carbamazepine-induced hiccups, as psychological factors can significantly influence symptomatology and treatment response. Patients experiencing persistent hiccups may develop anxiety, depression, or social withdrawal, exacerbating their overall distress. As a psychiatrist, I emphasize the importance of addressing psychosocial triggers and implementing holistic approaches to patient care. Cognitive-behavioral techniques, stress management strategies, and supportive counseling can complement pharmacological interventions, promoting patient resilience and coping mechanisms.”

## Discussion

The discussion surrounding carbamazepine-induced hiccups encompasses several key aspects, including the pathophysiology, clinical implications, management challenges, and potential strategies for optimizing patient care^1^. Understanding the complex interplay of factors contributing to hiccups associated with carbamazepine therapy is crucial for guiding clinical decision-making and improving treatment outcomes^3^. One of the major challenges associated with carbamazepine-induced hiccups is the lack of a definitive understanding of the underlying mechanisms^6^. While the exact pathophysiology remains elusive, several hypotheses have been proposed, including central nervous system (CNS) effects, alterations in neurotransmitter activity, and disruption of neural pathways involved in hiccup reflex arcs^7^. Carbamazepine’s anticholinergic properties, sodium channel blockade, and GABA receptor modulation may contribute to its potential hiccup-inducing effects^8^. However, further research is needed to elucidate the precise mechanisms and identify predictors of susceptibility to carbamazepine-induced hiccups. Clinical manifestations of carbamazepine-induced hiccups can vary widely among patients, ranging from mild and transient episodes to persistent or intractable bouts requiring intensive management.^10^ Various factors, including individual susceptibility, dosage regimen, concomitant medications, and underlying medical conditions, may influence the severity and duration of hiccups. Patients with epilepsy, psychiatric disorders, or neurological comorbidities may be at higher risk for developing hiccups or experiencing more severe symptoms, complicating their clinical course and treatment response^11^. Complications associated with carbamazepine-induced hiccups extend beyond physical discomfort, impacting patients’ quality of life, functional status, and treatment adherence^12^. Persistent or severe hiccups can disrupt patients’ sleep patterns, interfere with oral intake, and impair social interactions, leading to psychosocial distress and emotional burden. Healthcare providers must consider the holistic needs of patients affected by carbamazepine-induced hiccups, addressing their condition’s physiological and psychosocial aspects to optimize overall well-being^13^. Management of carbamazepine-induced hiccups poses several therapeutic challenges, as current treatment options are limited and often yield variable or suboptimal outcomes^14^. Pharmacological interventions, such as proton pump inhibitors (PPIs), antipsychotics, benzodiazepines, or dopamine receptor antagonists, may be used empirically to alleviate hiccups, although their efficacy remains uncertain^15^. Non-pharmacological approaches, including behavioral techniques, acupuncture, or diaphragmatic breathing exercises, may offer additional adjunctive benefits but require further investigation to determine their role in hiccup management^16^. Individualized treatment strategies are essential for tailoring interventions to patients’ needs, preferences, and underlying etiologies^1^. A multidisciplinary approach involving collaboration among neurologists, gastroenterologists, psychiatrists, and other healthcare professionals is paramount for optimizing patient care and addressing the multifaceted nature of carbamazepine-induced hiccups^17^. Shared decision-making, patient education, and ongoing monitoring are integral components of comprehensive hiccup management, fostering a patient-centered approach that prioritizes symptom relief, functional improvement, and overall quality of life^18^. Future directions for research in carbamazepine-induced hiccups should focus on elucidating the underlying pathophysiological mechanisms, identifying novel therapeutic targets, and evaluating the comparative effectiveness of different treatment modalities^19^. Large-scale prospective studies, randomized controlled trials, and systematic reviews are needed to establish evidence-based guidelines for hiccup management, inform clinical practice, and improve patient outcomes^20^. Additionally, efforts to raise awareness, promote interdisciplinary collaboration, and enhance patient support networks are essential for addressing the unmet needs of individuals affected by carbamazepine-induced hiccups and facilitating access to comprehensive care services^21^.

### Gaps and limitations in the existing literature

While the existing literature on carbamazepine-associated hiccups in epileptic patients provides valuable insights, it also exhibits several gaps and limitations that warrant further investigation. By critically examining these gaps, we can identify opportunities for future research and advancements in our understanding of this intriguing clinical phenomenon.

One significant area for improvement in the existing literature is the need for robust epidemiological data on the prevalence and incidence of carbamazepine-induced hiccups. Despite being a commonly prescribed antiepileptic drug, there is a lack of large-scale population-based studies examining the frequency and distribution of hiccups among carbamazepine users. Existing studies often rely on small sample sizes or retrospective case series, limiting the generalizability of findings and hindering our ability to assess the actual burden of this side effect^12^.

Moreover, the existing literature must be more consistent in study designs, methodologies, and outcome measures. There needs to be standardized approaches for assessing and diagnosing carbamazepine-induced hiccups, leading to heterogeneity in reported findings. Variations in inclusion criteria, duration of follow-up, and definition of outcomes further complicate comparisons across studies. Additionally, many studies rely on self-reporting or retrospective data collection methods, introducing potential biases and limitations in data accuracy^13,14^.

Furthermore, most existing literature consists of cross-sectional analyses or retrospective studies, lacking longitudinal follow-up to evaluate the long-term outcomes and trajectories of carbamazepine-associated hiccups. Longitudinal studies are essential for assessing hiccups’ persistence, resolution, or recurrence over time and identifying potential treatment response or prognosis predictors. The absence of longitudinal data limits our ability to make meaningful conclusions about the natural history and clinical course of carbamazepine-induced hiccups^15,16^.

Additionally, the existing literature often needs more comprehensive assessments of potential confounding factors or interactions that may influence the occurrence or severity of carbamazepine-associated hiccups. Factors such as concomitant medications, comorbid medical conditions, and lifestyle factors (e.g. smoking and alcohol consumption) may modulate individual susceptibility to hiccups but are frequently overlooked or underreported in existing studies. Please account for these confounders to ensure the validity and interpretability of study findings and avoid erroneous conclusions^9,10^.

Moreover, more studies are needed to investigate the psychosocial impact and quality-of-life implications of carbamazepine-induced hiccups on affected individuals. While hiccups may be perceived as a benign side effect, they can have significant psychosocial ramifications, including embarrassment, social stigma, and impaired daily functioning. However, few studies have systematically assessed the psychosocial burden of hiccups or explored patients’ experiences, coping strategies, and support needs. This gap in the literature highlights the importance of adopting a patient-centered approach and considering the holistic impact of hiccups on individuals’ well-being^11,12^.

### New knowledge and contributions

Carbamazepine, a cornerstone in the management of epilepsy, has long been recognized for its efficacy in controlling seizures. However, an intriguing side effect has emerged amidst its therapeutic benefits—carbamazepine-associated hiccups. This phenomenon, though relatively understudied, presents a fascinating intersection between pharmacology and neurophysiology, offering valuable insights into the complexities of drug-induced adverse reactions in epileptic patients.

A comprehensive review of the existing literature unveils a wealth of new knowledge and contributions to understanding carbamazepine-induced hiccups. Through meticulous synthesis and analysis, this review illuminates several vital insights and advances our comprehension of this unique clinical phenomenon.

One of this review’s primary contributions is identifying and exploring the mechanisms and pathophysiology underlying carbamazepine-induced hiccups. By synthesizing studies on neurotransmitter systems, neural oscillations, and pharmacogenetic factors, we gain a deeper understanding of the intricate interplay between pharmacological effects and neural circuits involved in the hiccup reflex. For example, studies by^1,2^ have highlighted the role of GABA and glutamate neurotransmitter systems in mediating the pharmacological effects of carbamazepine on neural circuits associated with hiccup generation^2,3^. Additionally, investigations into pharmacogenetic factors, such as genetic polymorphisms in drug-metabolizing enzymes, provide valuable insights into individual susceptibility to carbamazepine-induced hiccups^4^.

Furthermore, this review identifies risk factors and patient characteristics associated with carbamazepine-induced hiccups. Synthesis of epidemiological data and case studies reveals potential predictors such as age, dosage, and individual susceptibility. For instance, studies by^5,6^ have reported an increased incidence of hiccups with higher carbamazepine dosages and in older patients^5,6^. Moreover, genetic predisposition and underlying medical conditions may influence individual susceptibility, warranting personalized risk assessments and tailored treatment approaches^7^.

In addition to mechanistic insights and risk factor identification, this review provides valuable perspectives on management strategies for carbamazepine-induced hiccups. We comprehensively understand current approaches and their limitations by evaluating clinical guidelines and therapeutic interventions. For instance, while dosage adjustments and alternative antiepileptic agents may sometimes alleviate hiccups, their efficacy varies among patients^8,9^. Non-pharmacological interventions, such as breathing exercises and behavioral therapies, offer promising avenues for symptom management but require further investigation^10^. Furthermore, the review emphasizes the importance of a multidisciplinary approach to care involving neurologists, pharmacists, and mental health professionals to address both physiological and psychosocial aspects of hiccups in epileptic patients^9,10^.

Despite these advancements, the review also identifies gaps and limitations in the existing literature that warrant further investigation. For example, there needs to be more robust epidemiological data on the prevalence and incidence of carbamazepine-induced hiccups, hindering our understanding of the actual burden of this side effect. Additionally, there needs to be more consistency in study designs, methodologies, and outcome measures across existing literature, making comparing findings and drawing definitive conclusions challenging. Moreover, the lack of longitudinal studies examining the long-term outcomes and trajectories of carbamazepine-associated hiccups limits our ability to assess the persistence, resolution, or recurrence over time.

### Unique findings and perspectives

Exploring carbamazepine-associated hiccups in epileptic patients has unearthed several unique findings and perspectives that contribute to our understanding of this intriguing clinical phenomenon. Through meticulous analysis and synthesis of existing literature, we can identify novel insights and perspectives that shed light on the complexities of drug-induced adverse reactions and inform more effective management strategies.

One of the unique findings emerging from the literature is the potential role of pharmacogenetic factors in modulating individual susceptibility to carbamazepine-induced hiccups. Pharmacogenetics investigates how genetic variations influence drug response and metabolism, providing valuable insights into interindividual variability in drug effects. Studies by^1,2^ have highlighted the impact of genetic polymorphisms in drug-metabolizing enzymes, such as cytochrome P450 (CYP) enzymes, on carbamazepine metabolism and pharmacokinetics^1,2^. Variations in these enzymes may affect carbamazepine clearance rates, leading to differences in drug concentrations and susceptibility to adverse reactions, including hiccups. By considering pharmacogenetic factors, clinicians can personalize treatment approaches and optimize drug dosing regimens to minimize the risk of hiccups in susceptible individuals.

Moreover, the literature offers unique insights into the psychosocial impact and quality-of-life implications of carbamazepine-induced hiccups on affected individuals. While hiccups may be perceived as a benign side effect, they can have significant psychosocial ramifications, including embarrassment, social stigma, and impaired daily functioning. Studies by^3,4^ have highlighted the importance of adopting a patient-centered approach and considering the holistic impact of hiccups on individuals’ well-being^2^. Understanding patients’ experiences, coping strategies, and support needs is essential for providing comprehensive care and addressing the psychosocial burden of hiccups in epilepsy management. By integrating psychosocial assessments into clinical practice, healthcare providers can better support patients and improve their overall quality of life.

Furthermore, the literature offers unique perspectives on multidisciplinary management approaches for carbamazepine-induced hiccups. Recognizing the multifaceted nature of hiccups and their potential impact on various aspects of patients’ lives, clinical guidelines advocate for a collaborative care model involving neurologists, pharmacists, and mental health professionals. Studies by^5,6^ have highlighted the importance of addressing both physiological and psychosocial aspects of hiccups in epilepsy management^5,6^. This holistic approach encompasses pharmacological interventions, behavioral therapies, and psychosocial support services to address the diverse needs of affected individuals. By fostering interdisciplinary collaboration and communication, healthcare teams can develop tailored treatment plans that optimize patient outcomes and enhance overall care quality.

Additionally, the literature offers unique perspectives on the potential implications of carbamazepine-associated hiccups for pharmacovigilance and drug safety monitoring. Adverse drug reactions, including hiccups, are essential considerations in pharmacovigilance efforts aimed at monitoring the safety of antiepileptic drugs. Studies by^5,7^ underscore the significance of reporting and documenting adverse reactions to facilitate early detection and intervention^5,6^. By enhancing pharmacovigilance systems and raising awareness among healthcare providers and patients, we can improve the timely detection and management of hiccups and other adverse drug reactions, enhancing patient safety and optimizing treatment outcomes.

### How to apply this knowledge to routine clinical practice

Applying the knowledge gained from exploring carbamazepine-associated hiccups in epileptic patients to routine clinical practice is essential for optimizing patient care and outcomes. By integrating evidence-based recommendations and innovative approaches into clinical decision-making, healthcare providers can effectively manage hiccups and improve the overall quality of life for affected individuals.

One key recommendation for clinical practice is the adoption of personalized treatment approaches based on individual patient characteristics and risk factors. Pharmacogenetic considerations play a crucial role in optimizing drug therapy and minimizing the risk of adverse reactions, including hiccups. By assessing patients’ genetic profiles and considering factors such as CYP enzyme polymorphisms, healthcare providers can tailor carbamazepine dosing regimens to minimize the risk of hiccups in susceptible individuals^1,2^. Furthermore, monitoring serum drug concentrations and adjusting dosages based on TDM results can help optimize drug efficacy while minimizing adverse effects^5^.

In addition to pharmacogenetic considerations, clinical guidelines recommend a multidisciplinary approach to managing carbamazepine-induced hiccups involving neurologists, pharmacists, and mental health professionals. This holistic approach addresses hiccups’ physiological and psychosocial aspects and emphasizes the importance of patient-centered care^3,41^. Behavioral therapies, such as diaphragmatic breathing exercises and biofeedback techniques, can complement pharmacological interventions and help alleviate hiccup symptoms^5,7^. Moreover, psychosocial support services, including counseling and support groups, can provide individuals with coping strategies and emotional support to navigate the challenges associated with hiccups^5^.

Furthermore, healthcare providers should prioritize patient education and communication to empower individuals with the knowledge and skills to manage hiccups effectively. Educating patients about the potential side effects of carbamazepine, including hiccups, and guiding symptom recognition and management strategies can enhance medication adherence and improve treatment outcomes. Open and transparent communication between healthcare providers and patients fosters trust and collaboration, enabling shared decision-making and tailored treatment plans^5^.

Additionally, healthcare providers should remain vigilant in monitoring for the onset of hiccups in patients receiving carbamazepine therapy, particularly those at higher risk based on age, dosage, and genetic predisposition. Routine monitoring of adverse drug reactions through pharmacovigilance systems and adverse event reporting mechanisms is essential for early detection and intervention. Healthcare providers should promptly evaluate and manage hiccups by established clinical guidelines, considering alternative antiepileptic agents or adjunctive therapies if necessary^5,7^.

Furthermore, ongoing research and collaboration among healthcare professionals, researchers, and pharmaceutical companies are crucial for advancing our understanding of carbamazepine-associated hiccups and improving treatment strategies. Clinical trials investigating novel therapeutic approaches, such as targeted pharmacological agents or neuromodulation techniques, hold promise for addressing the underlying mechanisms of hiccups and developing more effective treatments^1,2^. By actively participating in research initiatives and contributing to evidence-based practice guidelines, healthcare providers can drive innovation and improve outcomes for individuals affected by carbamazepine-induced hiccups.

## Conclusion

Carbamazepine-induced hiccups represent a clinically significant side effect in a subset of epileptic patients receiving this medication. Despite its relatively low prevalence, hiccups can have substantial implications for patient well-being, including disruptions in sleep, impaired social functioning, and decreased quality of life. Effective management strategies, including dose adjustments, medication discontinuation, and adjunctive therapies, are essential for optimizing patient care and mitigating the impact of hiccups on daily life. Collaborative efforts among healthcare professionals, informed by a comprehensive understanding of the underlying pathophysiology, are crucial for achieving favorable treatment outcomes. Further research is warranted to elucidate the mechanisms underlying carbamazepine-induced hiccups and to explore novel therapeutic approaches, with the ultimate goal of improving patient outcomes and enhancing the management of epilepsy-related side effects.

## Ethics statement

The comprehensive review on carbamazepine-associated hiccups in epileptic patients adheres to ethical guidelines and principles governing research and publication integrity. All information presented in this review is based on previously published literature, research studies, and clinical guidelines, and no original data or human subjects were involved in preparing this manuscript. Therefore, institutional review board approval did not apply to this research endeavor.

The authors have complied with ethical standards regarding using copyrighted material, proper citation and attribution of sources, and avoiding plagiarism. Any images, figures, or tables included in this review are original creations or have been appropriately sourced and credited to the original authors and publications.

Furthermore, the authors have disclosed any conflicts of interest or financial relationships that may have influenced the content of this review. No funding or financial support was received for the preparation of this manuscript, and any potential biases or affiliations have been transparently disclosed.

## Consent

Informed consent was not required for this article due to the use of publicly available information and data, which was obtained and analyzed in an aggregated and de-identified manner. The study did not involve any direct interaction or intervention with human subjects, and the research findings were based solely on existing public knowledge and data sources. Therefore, no personal information or individual participation was involved, eliminating the need for informed consent.

## Sources of funding

The author declared no financial relationships at present or within the previous 3 years with any organizations that might have an interest in the submitted work.

## Author contribution

The author played important roles to actualize this article as shown below: C.E.: conceptualization, visualization, supervision, oversight and leadership, writing of the original draft.

## Conflicts of interest disclosure

In compliance with the ICMJE uniform disclosure form, all authors declare the following: Payment/services info: All authors have declared that no financial support was received from any organization for the submitted work.

## Guarantor

Chukwuka Elendu.

## Data availability statement

Data will be made available by the authors upon reasonable request.

## Provenance and peer review

Commissioned and externally peer-reviewed.

## Declaration/disclaimer/disclosure statement

The information presented in this comprehensive review on carbamazepine-associated hiccups in epileptic patients is intended for educational and informational purposes only. While every effort has been made to ensure the accuracy and reliability of the content, it is not intended to serve as medical advice or replace consultation with qualified healthcare professionals.

Readers are advised to consult their healthcare providers for personalized medical advice, diagnosis, and treatment recommendations tailored to their circumstances. This review’s authors, editors, and publishers disclaim any liability or responsibility for any adverse effects, complications, or consequences resulting from the information provided.

Furthermore, the views and opinions expressed in this review are those of the authors and do not necessarily reflect the official policy or position of affiliated institutions, organizations, or funding agencies. The authors, at this moment, disclose any conflicts of interest or financial disclosures related to the content of this review by ethical guidelines and professional standards.

Readers are encouraged to evaluate the information presented critically and consider alternative perspectives and sources of evidence. This review is not intended to endorse any specific products, treatments, or interventions, and readers are advised to exercise caution and discretion in interpreting and applying the information provided herein.
